# Comparative Analysis of Characteristic Volatile Compounds in Five Types of Infant Nutrition Powders by HS-GC-IMS and GC-MS

**DOI:** 10.3390/foods13050648

**Published:** 2024-02-21

**Authors:** Zhihua Yang, Jinjin Li, Xiaoming Guo

**Affiliations:** 1 Shenzhen Institute of Standards and Technology, Shenzhen 518033, China; 2 Shenzhen Key Laboratory of Food Nutrition and Health, College of Chemistry and Environmental Engineering, Institute for Innovative Development of Food Industry, Shenzhen University, Shenzhen 518060, China

**Keywords:** infant nutritional powder, HS-GC-IMS, volatile organic compounds, fatty acids, PLS-DA

## Abstract

This study employed the headspace-gas chromatography-ion migration spectrum (HS-GC-IMS) in conjunction with the gas chromatography-mass spectrometer (GC-MS) technique for the assessment of the flavor quality of complementary food powder intended for infants and young children. A total of 62 volatile compounds were identified, including aldehydes, esters, alcohols, ketones, pyrazines, and furans, among which aldehydes were the most abundant compounds. Based on the principal component analysis (PCA) and partial least squares discriminant analysis (PLS-DA) models, infant nutritional powder (YYB) from different manufacturers could be clearly distinguished. Among them, 2-hydroxybenzaldehyde, 1, 2-dimethoxyethane, 2-isobutyl-3-methoxypyrazine, and methyl butyrate were the four most critical differential volatiles. In addition, these differences were also manifested in changes in fatty acids. The reason for this phenomenon can be attributed to the difference in the proportion of raw materials used in nutrition powder, micronutrient content, and the packaging process. In conclusion, this study provides comprehensive information on the flavor quality of YYB, which can be used as a basis for quality control of YYB.

## 1. Introduction

Malnutrition in early childhood in China is a moderate public health problem [1]. After 6 months of age, the nutritional elements obtained from breast milk can no longer meet the healthy development needs of infants and young children, so semi-solid and solid foods need to be added in a timely and scientific manner, and gradually converted into domestic foods [2]. In China, the availability and affordability of nutrient-rich complementary foods are limited in many lower-income areas, mainly poor and less-educated rural areas, leading to widespread early stunting in infants and young children [3,4]. Since 2012, with the financial support of the central government, the National Health Commission and the All-China Women’s Federation have launched a nutrition improvement project for children in poor areas. Infant nutrition powder can be used for nutritional intervention in children in underprivileged areas to lower the risk of infant malnutrition, according to China’s National Nutrition Plan 2017–2030. Infant Supplemental Nutrition Pack (YYB), is a supplement containing a variety of micronutrients (vitamins, minerals, etc.) added to ready-to-eat complementary foods for infants and toddlers (6–36 months), and can also be used as a dietary supplement for children (37–60 months) for their growth needs [5].

As awareness of children’s nutrition increases and the demand for infant nutritional supplements grows, related technologies, policies, and regulations are gradually maturing. This has led to the emergence of various brands and formulations of products. Complementary food nutrition packaging products are one of the products implemented under the policy, and have been widely used in poor rural areas and areas affected by natural disasters for nutrition improvement in 6–24-month-old infants. At present, there are a variety of infant complementary food nutrition products on the market, due to the difference in nutritional composition, trace elements, and processing technology, the quality of YYB is also different, resulting in consumer liking being inconsistent [6]. As a complementary food for infants and young children, the product is suitable for the preferences of infants and young children, which is a prerequisite to ensure that the nutrients are effectively supplemented. In the practical application of nutrition packages, it is not uncommon for children and parents to refuse to eat because of “odor” and other reasons, which is very unfavorable to the effective implementation of nutrition packages and the implementation of nutrition improvement for children in poor areas, but measures can be taken to effectively improve the problem [7].

The flavor of nutrition packages is a key factor in determining whether consumers buy and eat them. Volatile compounds, including aldehydes, ketones, and alcohols, which can originate from the primary and secondary oxidation products of unsaturated fatty acids, are integral in forming off-flavors that ultimately affect the shelf life and storage resilience of food products [8]. Therefore, it is very important to clarify the composition of volatile flavor substances in infant complementary food nutrition powder and the flavor differences among different nutrition packages. With the development of volatile compound detection technology, gas chromatography-ion mobility spectrometry (GC-IMS), an emerging detection technology, has both GC separation characteristics and IMS high sensitivity and does not require sample pretreatment [9]. It has been increasingly applied to the research of the flavor and quality of various foods such as nutritional powders [5].

Although researchers have explored the processing technology and flavor of nutrition powder, there are few reports on the differences in the flavor of infant complementary food powder products from different manufacturers. Therefore, we randomly selected five types of YYB with relatively high market shares from the Chinese market and used GC-IMS technology combined with gas chromatography-mass spectrometer (GC-MS) technology to detect flavor compounds and fatty acids (FAs) in YYB. The volatile flavor substances in YYB manufactured by different companies were compared using multivariate statistical analysis. Meanwhile, multivariate statistical analysis was also performed to identify the characteristic volatile flavor substances of YYB products. The findings of this research could provide a significant theoretical foundation for quality control of infant complementary food nutrition powder.

## 2. Materials and Methods

### 2.1. Materials and Chemicals

According to the market share of infant nutritional powders in China, five products (denoted as numbers 1–5, respectively) with high market share were selected for analysis. The production of YYB involves selecting raw materials, implementing quality control, adding excipients, mixing, and packaging, adhering to the Chinese national food safety standard GB 22570–2014 for infant food supplements. The 5 YYB samples contained protein (>25%) and fat (~10%), complemented by a modest amount of vitamins and minerals to support infant nutrition. All YYB products were placed in the LHS-250SC constant temperature and humidity incubator (Shanghai Yiheng Scientific Instrument Co., Ltd., Shanghai, China) for 90 days of accelerated storage. The storage temperature was 37 ± 2 °C, and the relative humidity (RH) was 75%. For simplicity in notation, products are designated with a suffix “A” after the YYB number to denote the condition at day 0 of storage (e.g., YYB-1A), and a suffix “B” to represent the state following 90 days of storage (e.g., YYB-1B).

### 2.2. Color Measurements

Color measurements were obtained using an Ultra Scan PRO colorimeter (Hunter Lab, USA), through pouring the powder sample into a transparent, self-sealing bag and calibrating against a standard white board, as previously described by Manzi et al. [10]. The CIE L* a* b* system, which describes the change in lightness from black (0) to white (100) with L*, and the red (+a*) to green (−a*) color spectrum with a*, and the yellow (+b*) to blue (−b*) color spectrum with b*, was employed to characterize the color. ΔE and W values were calculated, respectively, as follows:

W = 100 − [(100 − L*)^2^ + a*^2^ + b*^2^]^1/2^

$$\Delta E=\sqrt{(L^{*}-L{)}^{2}+(a^{*}-a{)}^{2}+(b^{*}-b{)}^{2}}$$

where W value was the white value; L*, a*, and b* were the measured values of the sample. ΔE was the total color difference value.

### 2.3. Headspace (HS)-GC-IMS Analysis

The volatile organic compounds (VOCs) of the sample were determined for different accelerated storage times (0 or 90 days) using the GC-IMS (FlavorSpec^®^) (GAS, Dortmund, Germany), following the method of Li et al. with some modifications [9]. Two grams of YYB were placed into a headspace glass vial and incubated at 60 °C for 20 min, followed by automatic injection of 800 μL using a heated syringe set at 85 °C. The analysis time was set to 30 min, with a chromatographic column (MXT-5) measuring 15 m in length, 0.53 mm in diameter, and 1 μm in thickness. The column temperature was maintained at 60 °C, while the IMS temperature was set to 45 °C. Nitrogen gas (N_2_) was used as the carrier gas. The flow rate started at 2 mL/min for the first 2 min, increased to 10 mL/min for the next 10 min, then to 100 mL/min for another 10 min, and finally reached 150 mL/min for the last 20 min to stop the flow. External references, N-ketones C4–C9 (Sinopharm Chemical Reagent Beijing Co., Ltd., Beijing, China), were utilized for calculating the retention index (RI) of VOCs. The signal intensity of compounds in LAV software (version 2.2.1) was converted into peak volume comparisons to quantify the relative variation difference of VOCs.

### 2.4. Analysis of Fatty Acids in YYB Samples from Different Manufacturers before and after Storage Using GC-MS

FAs in YYB samples from various manufacturers were analyzed according to the method (with minor modifications) of Walczak et al. [11]. GC (6890N, Agilent Technologies, Waldron, Germany) and MS (Agilent 5975 Inert XL MSD, Zebron ZB-WAX capillary column, 30 m long, 0.25 μm inner caliber; Phenomenex, Torrance, CA, USA) were utilized for the FAs in YYB samples GC-MS analysis. The column temperature underwent a programmed increase, starting at 60 °C for 2 min, followed by a rise to 150 °C at a rate of 13 °C/min, and finishing with an increase to 230 °C at 2 °C/min, with a hold for 6 min. Meanwhile, the splitless injector temperature was fixed at 240 °C. The mass spectrometry analyses were performed in full-scan mode, scanning across a mass range of 35 to 450 *m*/*z*. The electron energy was 70 eV, and both the ion source and line transfer were held at 200 °C. Chromatographic data were obtained using Agilent Chromatography Workstation 3 software. The identification of FAs in YYB samples was based on retention time comparison with standard and spectral analysis.

### 2.5. Statistical Analysis

Differences among YYB were analyzed using analysis of variance (ANOVA) and Duncan’s multiple range test with SPSS 19.0 software (SPSS Inc., Chicago, IL, USA). VOCs were searched through the Hanon 3H_IMS_2022 database (Beijing, China) and qualitatively identified by retention time reference values. All experiments were repeated three times, and the significance was evaluated at a confidence level of ±5% (*p* < 0.05). Principal component analysis (PCA) and partial least squares discriminant analysis (PLS-DA) were conducted using SIMCA-P software (Umetrics, version 13.0, Malmö, Sweden), MetaboAnalyst 5.0 software (http://www.metaboanalyst.ca, accessed on 10 May 2023) and networking tool (https://www.chiplot.online/#Pie-plot, accessed on 10 May 2023). GraphPad Prism (version 9.5.0; GraphPad Software, Boston, MA, USA) and PowerPoint (Microsoft Office 365; Redmond, WA, USA) were used to generate the remaining figures. All results were expressed as the mean ± standard deviation.

## 3. Results and Discussion

### 3.1. The Color Changes of Five Types of Infant Nutrition Powder after Accelerated Storage

**Figure 1:**
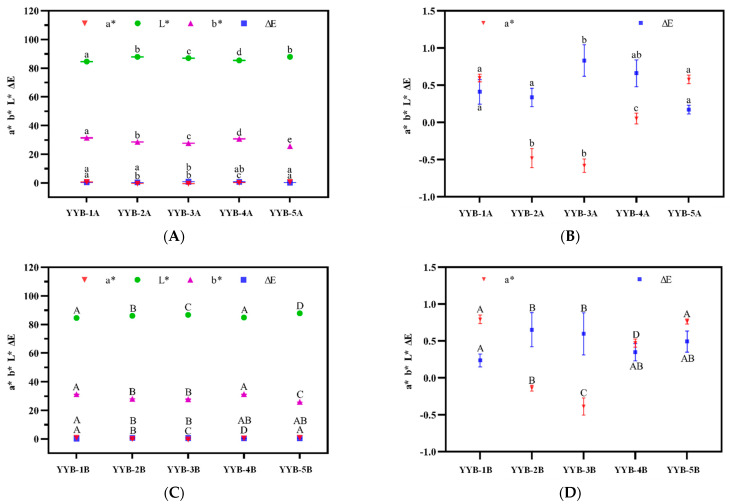
The color changes with different manufacturers of YYB after accelerated storage. (**A**): YYB stored for 0 days; (**B**): partial enlargement of (**A**); (**C**) YYB stored for 90 days; (**D**): partial enlargement of (**C**). Different letters above the bars indicate that the samples exhibit statistically significant differences (*p* < 0.05) from one another. Uppercase letters represent differences among samples on day 0, while lowercase letters denote differences among samples stored after 90 days.

The color is an important test index of powder product quality, which directly affects consumers’ satisfaction with the product. Measuring the L* a* b* values of YYB can be used to quantify the difference in perceived color intensity of the sample under accelerated storage time. As can be seen from Figure 1, when stored for 0 days, the values of ∆E, L*, and b* of samples from different manufacturers have no significant difference overall. After three months of storage, the value of ∆E shows an upward trend, the value of L* shows an upward trend, the value of b* shows a downward trend, and the value of a* has no obvious change. It showed that the color of nutrient powder became dark yellow after storage. Studies have shown that egg white powder has a similar color change during storage [12]. After 90 days of accelerated storage, due to the oxidation and deterioration of proteins in the sample components, the spatial configuration was destroyed and hydrophobic pores were formed, resulting in a slight increase in L* value [13]. In addition, during storage, the Maillard reaction products in YYB form brown aggregates, which change the color appearance of the sample [14,15]. The relatively high storage humidity (75% RH) fosters microbial activity in nutrient powders, enhancing the hydrolysis of proteins and starches. This generates additional amino and carbonyl groups, accelerating the Maillard reaction and increasing yellow compound content, resulting in significant alterations to the L* and b* values of the powder [16]. After 90 days of accelerated storage, the color difference of YYB-1 and YYB-5 did not change significantly, which may be due to the high nitrogen content when the product was filled, which effectively protected the stability of the color. The changes in the L* values in YYB-3 and YYB-5 may be due to the smaller particles in the nutrient powders, which have a larger specific surface area, resulting in a larger reflection factor for the powders [17]. The YYB was filled with nitrogen during the packaging process, which also plays a certain protective role in the storage process. The difference between YYB from different manufacturers was related to the amount of nitrogen filled in the packaging and the difference between the packaging materials. At the same time, if the product needs long-term storage, it should be carried out in a dry and low-temperature environment.

### 3.2. Changes in Volatile Compounds in Infant Nutrition Powder from Different Manufacturers

**Figure 2:**
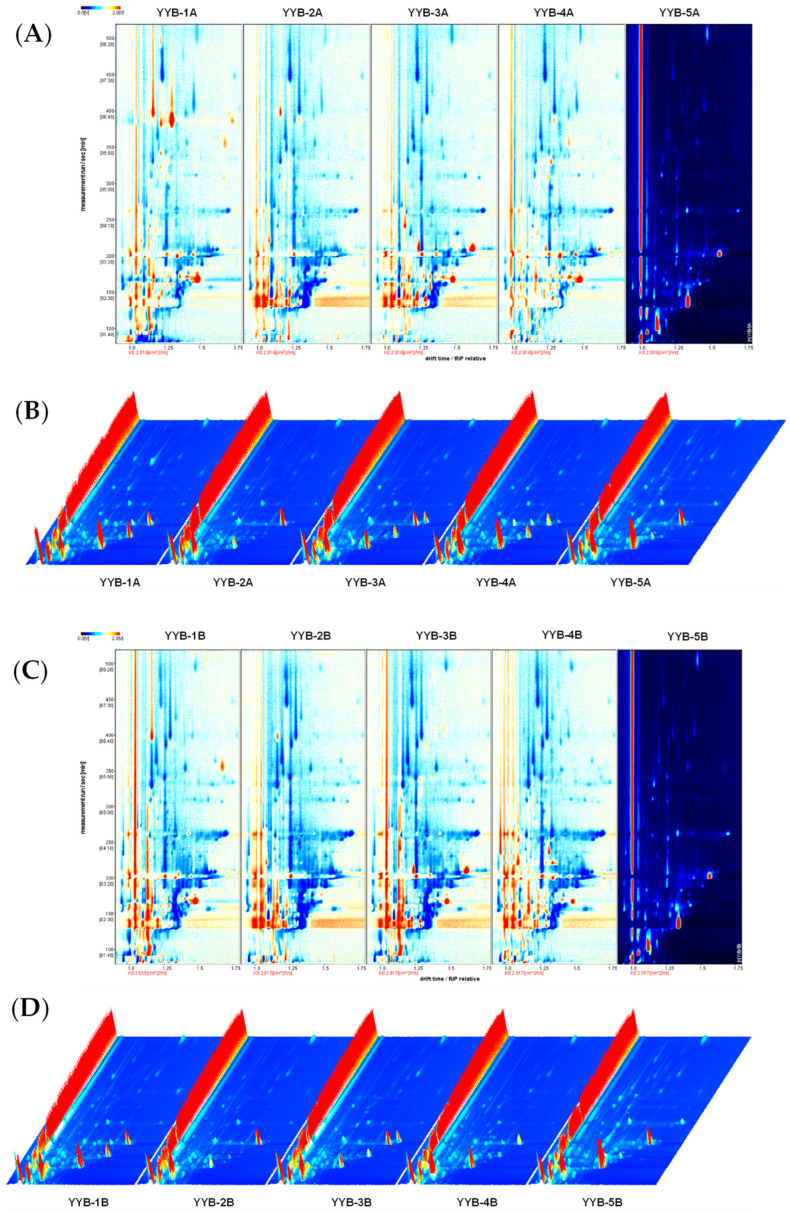
The two-dimensional topographic plots of flavor compounds in YYB under accelerated storage conditions. (**A**) The YYB under the condition with 37 °C and 75% RH stored for 0 days, (**C**) The YYB under the condition with 37 °C and 75% RH stored for 90 days. Three-dimensional topographic plots and chromatograms of flavor compounds in YYB under accelerated storage conditions. (**B**) The YYB under the condition with 37 °C and 75% RH stored for 0 days, (**D**) The YYB under the condition with 37 °C and 75% RH stored for 90 days.

**Figure 3:**
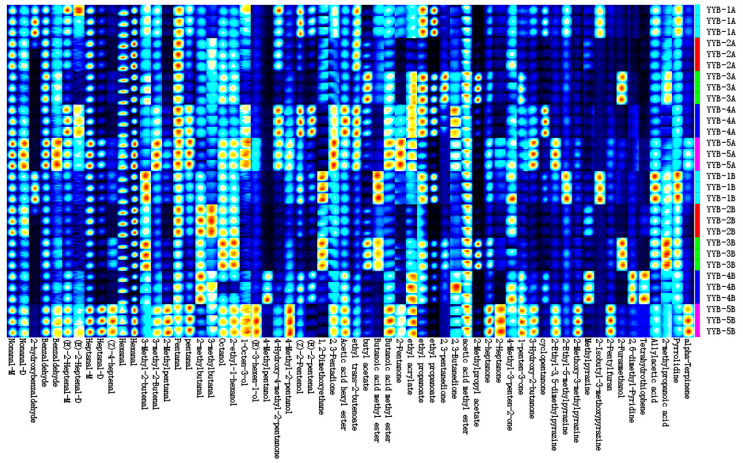
The gallery plots of flavor compounds in nutrition powder (YYB) under accelerated storage conditions. The YYB was stored at 37 °C and 75% RH for 90 days. Numbers represent different manufacturers; letters represent storage time.

This study employed HS-GC-IMS to analyze VOCs in nutrition powder during accelerated storage. HS-GC-IMS generated a 3D topographic plot to display the statistical data, revealing diverse peak intensities among the VOCs in different samples. The 3D topographic plot employed the Y axis to represent the retention time of the gas chromatograph, the X axis to indicate ion migration time for identification, and the Z axis to quantify peak values. Each peak corresponds to a specific volatile compound, with color indicating its intensity. Red peaks symbolized VOCs exhibited higher signal intensities. The findings revealed the presence of various VOCs in YYB. The peak signal intensity and signal intensity of VOCs in YYB produced by different manufacturers are different. As shown in Figure 2, a total of 62 VOCs were identified in five types of infant nutrition powders including 19 aldehydes, 13 esters, 11 alcohols, 6 ketones, 5 pyrazines, 2 furans, 2 acids, and 4 other compounds (Table 1). Most of the VOCs in five types of YYB products correspond to different signal strengths, indicating that YYB of different manufacturers has its own characteristics. To better understand the VOC differences between different samples, we classified the VOC of YYB in the fingerprint (Figure 3).

A deeper red color indicated a higher concentration of the compound. White indicated less concentration of the volatile compound, whereas a volatile compound may produce more than a signal or spot (monomer, dimer or even trimer). Each row represented a sample, and every column represented a signal peak. Characteristic volatile flavor compounds and differences in samples can be clearly seen in the species fingerprint.

Aldehydes and ketones mainly come from the oxidative degradation of fats and are closely related to changes in food flavor [18]. Nonanal monomer, benzaldehyde, heptanal, hexanal, and pentanal were found to have stronger signal intensities in YYB products from different manufacturers, and these small-molecule aldehydes are one of the sources of floral and fruity aromas in YYB products [19]. The signal intensities of (E)-2-Heptenal, 3-Methyl-2-butenal, and 2-methylbutanal were higher in YYB-1, YYB-3, and YYB-5 products. Although Nonanal, heptanal, pentanal, and other aldehydes have a stronger signal intensity in YYB, the content of these compounds in the products of different manufacturers is still different, which indicates that the origin of the raw materials and the processing method may affect the compound types and content in the products [20]. During the accelerated storage process, the protein and fat in the nutritional powder undergo lipid oxidation and the Maillard reaction, which are prone to oxidation and decomposition, resulting in the release of free fatty acids. Saturated fatty acids are easily degraded into aldehydes, alcohols, ketones and other volatiles after being oxidized into unstable hydroperoxides, which are the precursors of aromatic compounds [21]. 2-methyl-butenal and 3-methyl-2-butenal were volatile aldehydes that were products of the metabolic degradation of fats or produced by the breakdown of proteins in raw materials [22]. At the same time, these flavor compounds played a crucial role in the formation of nutritional powder flavor [23]. Low concentrations of aldehydes usually have a grassy taste and sweetness, while high concentrations may produce an odor [24].

Alcohols were flavor substances formed by aldehyde reduction, amino acid metabolism, or lactose fermentation, with polyunsaturated fatty acids as precursors. The formation of alcohols is closely related to the oxidation of fats and the reduction of ketones. 4-methyl-2-pentanol, (E)-2-pentenal, 1,2-dimethoxyethane have a strong signal strength in YYB-4A. 1-Octen-3-ol, 4-hydroxy-4-methyl-2-pentanone, 1, 2-dimethoxyethane were stronger in YYB-1A and YYB-5A, while octanol and 2-ethyl-1-hexanol were stronger in YYB-3A. The threshold for saturated alcohols is relatively high, and unsaturated alcohols such as 1-octen-3-ol, (E)-3-hexen-1-ol, (Z)-2-pentenol, (E)-2-pentenal provide fresh grassy and mushroom flavors [25]. 2-ethyl-1-hexanol, (E)-2-pentenal, (Z)-2-pentenol have a nutty aroma that will add to the grassy aroma of the powder [26]. After accelerated storage, the signal strength of octanol and 2-ethyl-1-hexanol in YYB-3B and YYB-5B was significantly enhanced, and the signal strength of 1-octen-3-ol and (E)-3-hexen-1-ol was stronger in YYB-5B, which may have given the products a unique flavor that was different from other products. The difference in the ratio of saturated alcohols/unsaturated compounds in the products may be one of the reasons for this difference. Both lipid oxidation and thermal degradation of proteins produce alcohol compounds [27], which also affect the flavor of nutritional powders [28].

The most ester compounds were detected in YYB-3 and YYB-4, including butyl acetate ethyl acrylate, ethyl propanoate, ethyl propanoate and 2,3-pentanedione. Acetic acid, hexyl ester, ethyl trans-2-butenoate, ethyl acrylate, and ethyl propanoate are found primarily in YYB-1. YYB-5 was dominated by butanoic acid methyl ester, 2-pentanone and ethyl acrylate. Hexyl acetate, butyl acetate, 2-methylpropyl acetate, butanoic acid methyl ester and ethyl acrylate mainly present fruity and woody aromas, which were one of the main aromas of nutrition powder. Ethyl acrylate is an important odorant with a strong signal strength and low threshold in YYB-1, 3 and 4. After accelerated storage, the signal intensity of butanoic acid methyl ester in YYB-1 and YYB-3 noticeably strengthens, likely as a result of the elevated content of alcohols and carboxylic acids. Moreover, the signal intensity of most ester compounds in different products is weakened to different degrees, showing a decrease in aromatic substances.

The signal intensity of heterocyclic compounds in the samples increased noticeably post-storage as compared to pre-storage. The heterocyclic nitrogen-containing compounds, including 2-methoxy-3-methylpyrazine, 2-isobutyl 3-methoxypyrazine, and 2-ethyl-5-methylpyrazine, were derived from nonenzymatic protein–sugar interactions [29]. The formation of 2-ethyl-3,5-dimethylpyrazine in dairy products is attributed to the Maillard reaction [30]. The augmentation in the production of nitrogen-containing compounds arising from the catabolic metabolism of proteins and free amino acids in nutritional powders indicates that nutritional powder products are not suitable for storage in high-temperature conditions, which puts forward requirements for storage of commercially available products [31].

### 3.3. Principal Component Analysis (PCA)-Based Fingerprint Similarity Analysis

**Figure 4:**
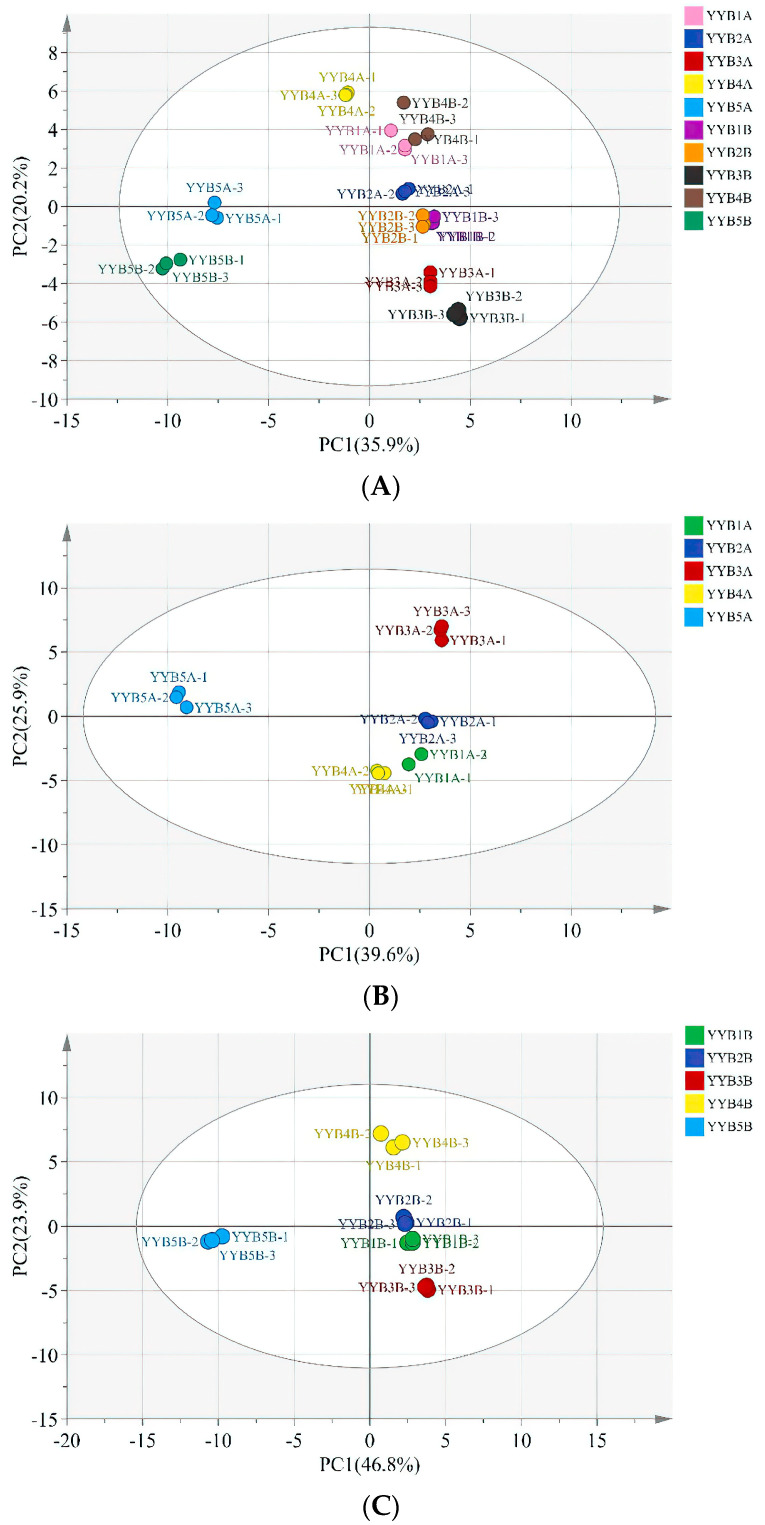
The principal component analysis (PCA) scatter diagram of flavor compounds in nutrition powder (YYB) under accelerated storage conditions. (**A**) The YYB under the condition with 37 °C and 75% RH stored for 0 days and 90 days, (**B**) the YYB under the condition with 37 °C and 75% RH stored for 0 days, (**C**) the YYB under the condition with 37 °C and 75% RH stored for 90 days.

To investigate the variation in VOCs among nutritional powders from various manufacturers, a PCA was conducted, as illustrated in Figure 4. The total variance contribution of PC1 (36.00%) and PC2 (20.00%) accounts for 56.00%. Based on the contribution rates of the principal component factors in the various samples, an evaluation of the patterns, similarities, and differences among the samples was conducted [32].

On the whole, the position of VOCs in the samples after accelerated storage is in the same quadrant as before storage, but with a certain distance [33]. The results indicated a change in the content of VOCs in the nutrient powder after accelerated storage. There was a significant difference between sample YYB-5 and the other four products. The sample points for the remaining four products are situated on the right side of the PCA diagram. The sample points gradually shifted to the left as the storage time increased. Nutrient powder in accelerated storage for 90 days and 0 days varies in the content and composition of compounds contained in samples.

**Figure 5:**
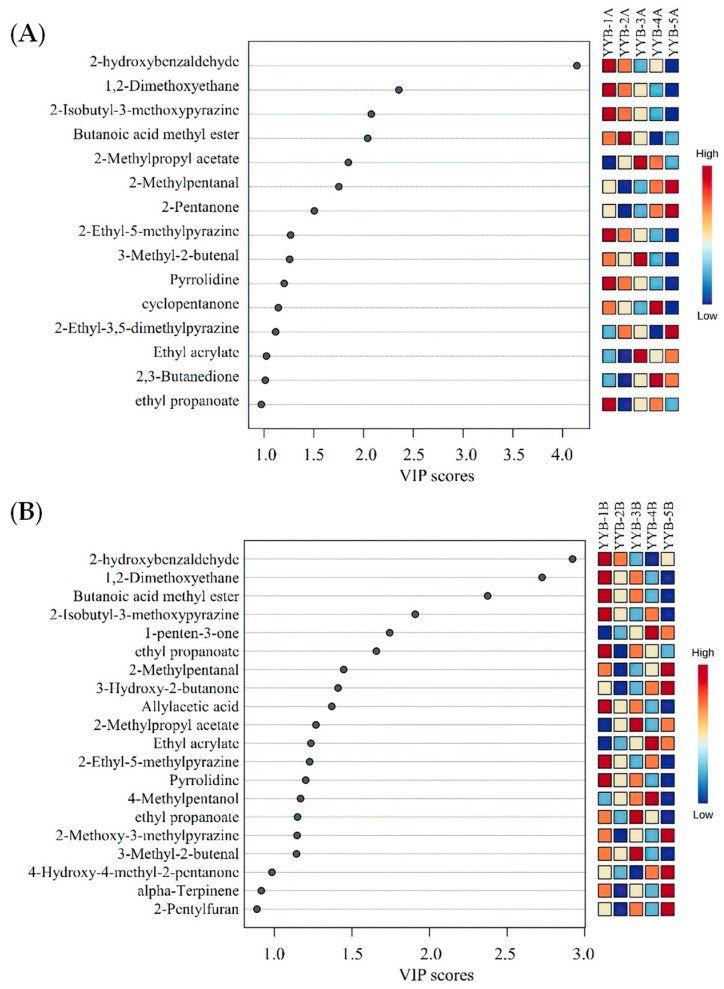
Variable importance in projection (VIP) scores of each variable in five types of YYB based on GC-IMS (VIP > 1, *p* < 0.05), (**A**): 0 d; (**B**): 90 d.

Based on the HS-GC-IMS data, a total of 10 qualitative differential characterization markers with variable importance in projection (VIP) >1 were screened (Figure 5), including four esters, three aldehydes, and three heterocyclic compounds. YYB-1 contained higher levels of 2-hydroxybenzaldehyde, YYB-3 contained higher levels of 2-methylpropyl acetate, 3-methyl-2-butenal and ethyl acrylate, and YYB-4 contained higher levels of Ethyl acrylate and 2,3-Butanedione. These VOCs can be used as potential markers to distinguish between the five different YYBs.

### 3.4. The Difference in Fatty Acid after Accelerated Storage of YYB from Different Manufacturers

FAs serve various roles in the nutritional package. In addition to providing stable and long-lasting energy, they are crucial components in cell membrane construction [34]. Table 2 and Table 3 display the results of FAs analysis conducted on YYB samples from various manufacturers using GC-MS. As shown in Table 2, a total of 19 FAs were detected in all unstored YYB samples. The number and species of FA varied among the groups; the YYB-1A group had 19 species, YYB-5A had 17, YYB-3A and YYB-4A had 15, and YYB-2A had 14. It is worth noting that changes in FA species were observed in some samples after 90 days of storage. Compared to the initial storage status, the absence of decanoic and myristic acids was observed in YYB-1B; YYB-3B showed an increase in myristate acid and docosahexaenoic acid; YYB-5B exhibited an augmentation in eicosatetraenoic acid and docosahexaenoic acid levels. Unlike the other groups, no significant alterations in FAs species were detected between the pre-storage and post-storage states in groups YYB-2 and YYB-4. Moreover, the concentrations of different FAs showed significant disparities (*p* < 0.05) among each YYB sample, both prior to and following storage.

**Figure 6:**
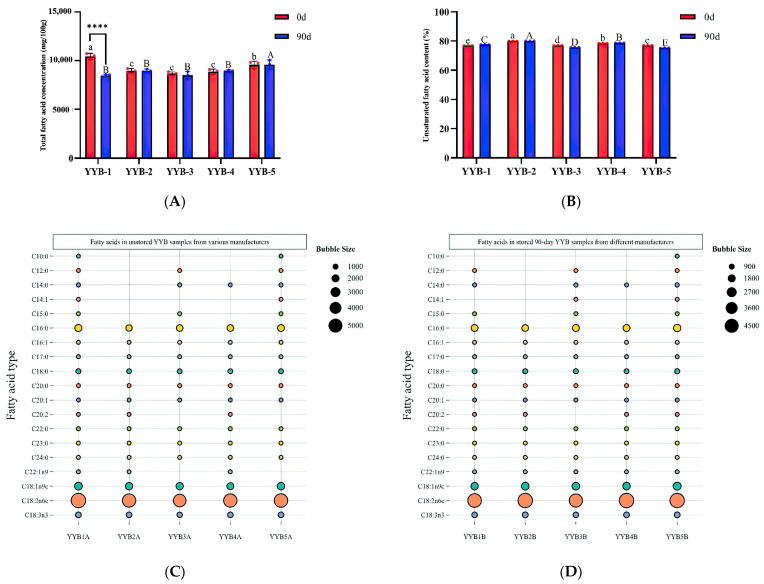

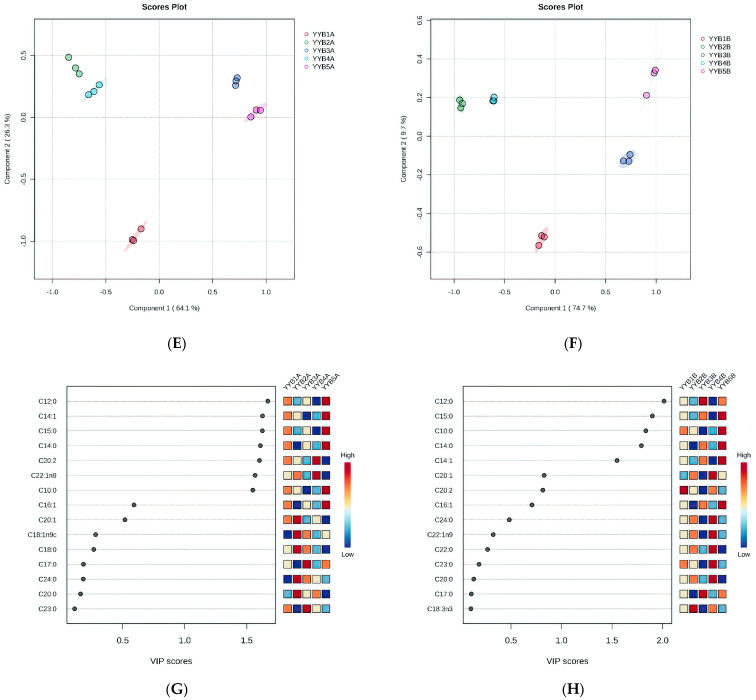
(**A**): Total fatty acid content in different YYB; (**B**): the content of unsaturated fatty acids in different YYB; In panels (**A**,**B**), samples are distinguished by different letters above the bars, showing statistically significant differences (*p* < 0.05) between them; uppercase letters represent differences among day 0 samples, while lowercase letters denote differences among day 90 samples; In panels (**A**), the asterisks **** represent statistical significance at the levels of *p* < 0.0001; (**C**): bubble chart of fatty acid content in different YYB storage for 0 days; (**D**): bubble chart of fatty acid content in different YYB stores for 90 days; (**E**): PLS-DA score chart of fatty acid content in different YYB storage for 0 days; (**F**): PLS-DA score chart of fatty acid content in different YYB storage for 90 days; (**G**): VIP chart of different YYB storage for 0 days; (**H**): VIP chart of different YYB storage for 90 days.

The total FAs concentration of each YYB sample is shown in Figure 6A. Significant changes in FAs concentrations were not observed between before and after storage for the four YYB groups except for the YYB-1 group. The FAs concentration of the YYB-1B group was significantly lower than that of the YYB-1A group (*p* < 0.001). After storage, the FAs concentration of the YYB-5B group was significantly higher (*p* < 0.05) when compared with the remaining four groups. Generally, moderate consumption of unsaturated fatty acid (UFA) yields several advantageous effects on the human body, including the promotion of heart health, support for the brain and nervous system, and facilitation of fat-soluble vitamin absorption [35]. Figure 6B illustrates that the UFA contents of the YYB-1B, YYB-2B, and YYB-4B groups exhibited significant increases compared to their pre-storage levels (*p* < 0.05), whereas the YYB-3B and YYB-5B groups experienced significant decreases (*p* < 0.05). This corresponds to the decline in acid compounds and the corresponding increase in aldehydes and esters in the GC-IMS outcomes.

It becomes evident that the dominant fatty acids in the five groups of YYB samples (Figure 6C,D) were palmitic acid, Omega-9 Oleic acid, Omega-6 (ω-6) Linoleic acid and Omega-3 (ω-3) α-linolenic acid, both before and after storage, of which Omega-6 (ω-6) Linoleic acid had the highest concentration, accounting for approximately 65% of the total UFA concentrations in the YYB samples before and after storage. It was the most dominant UFA in the YYB samples.

The PLS-DA method was employed to visualize the variations in fatty acid composition among YYB samples from different manufacturers. Additionally, the obtained VIP values facilitated the identification of significant differences in fatty acid profiles among the samples [36]. Before analysis, the data were normalized using probability quotient normalization (PQN) with the YYB-1A and YYB-1B groups as references, followed by a Log10 transformation. Figure 6E illustrates a distinct separation of the YYB sample groups before storage on the PLS-DA score plot, indicating differentiation among the five groups. The distance between the sample points on the scoring graph indicates the level of similarity or difference in their fatty acid compositions; a shorter distance corresponds to higher similarity, while a longer distance indicates lower similarity [37]. The YYB-2A and YYB-4A groups were positioned closely, located in the upper left portion of the score graph. Similarly, the YYB-3A and YYB-5A groups were positioned closely, located in the upper right portion of the score graph. Figure 6G presents the seven key differential fatty acids (VIP > 1) found among the five groups of YYB samples before storage, as depicted in the figure. These fatty acids include capric acid, lauric acid, myristic acid, myristoleic acid, pentadecanoic (alkyl) acid, eicosadienoic acid, and Omega-9 fatty acids. Based on the results of Figure 6G, an interesting phenomenon can be found, which was, except for the YYB-3B group, the positions of the other four groups of YYB samples on the scoring graphs did not change significantly. Only the position of YYB-3B group changed significantly, suggesting that the fatty acid composition and content of YYB-3 group samples significantly varied before and after storage. From Figure 6H, it can be observed that there were five key differential FAs in YYB samples after storage. They were capric acid, lauric acid, myristic acid, myristoleic acid, and pentadecanoic acid.

## 4. Conclusions

In this study, HS-GC-IMS technology, combined with GC-MS was used for the determination of the flavor quality of infant complementary food powder. A total of 62 volatile components, such as aldehydes, esters, alcohols, ketones, pyrazines, and furans were identified by HS-GC-IMS. Among them, aldehydes are the most abundant and have a low threshold, making them the most important flavor substances in the powder. Based on the PCA model, the nutritional package products of different manufacturers can be clearly distinguished. Significant variations in VOCs in the final product were attributed to differences in the ratio of raw materials, the addition of trace elements, and packaging processes. Furthermore, these differences were also reflected in the changes in fatty acids, with lauric acid and myristic acid serving as important indicators for distinguishing YYB from various manufacturers. In summary, this study provides comprehensive insights into the flavor quality of infant complementary food nutrition powder, which can provide a basis for the quality control of YYB.

## Figures and Tables

**Table 1:** Flavor compounds from YYB during storage.

No.	Compound	CAS	Formula	MW	RI ^1^	Rt ^2^	Dt ^3^
1	nonanal (M)	124-19-6	C_9_H_18_O	142.2	1107.9	506.703	1.94623
2	nonanal (D)	124-19-6	C_9_H_18_O	142.2	1108.2	507.28	1.47979
3	2-hydroxybenzaldehyde	90-02-8	C_7_H_6_O_2_	122.1	1041.5	400.178	1.16322
4	benzaldehyde (M)	100-52-7	C_7_H_6_O	106.1	962.9	311.291	1.14982
5	benzaldehyde (D)	100-52-7	C_7_H_6_O	106.1	963.3	311.572	1.46808
6	(E)-2-heptenal (M)	18829-55-5	C_7_H_12_O	112.2	958.6	307.64	1.25807
7	(E)-2-heptenal (D)	188255-5	C_7_H_12_O	112.2	959.9	308.764	1.67159
8	heptanal (M)	111-71-7	C_7_H_14_O	114.2	901.7	263.425	1.333
9	heptanal (D)	111-71-7	C_7_H_14_O	114.2	900.4	262.499	1.69684
10	(Z)-4-heptenal	6728-31-0	C_7_H_12_O	112.2	888.5	254.841	1.63226
11	hexanal (M)	66-25-1	C_6_H_12_O	100.2	783	206.799	1.26096
12	hexanal (D)	66-25-1	C_6_H_12_O	100.2	776.9	203.497	1.56295
13	3-methyl-2-butenal	107-86-8	C_5_H_8_O	84.1	781.1	205.79	1.35933
14	2-methylpentanal	123-15-9	C_6_H_12_O	100.2	748.4	188.78	1.51291
15	pentanal (M)	110-62-3	C_5_H_10_O	86.1	691.7	162.582	1.18891
16	pentanal (D)	110-62-3	C_5_H_10_O	86.1	690.6	162.115	1.42669
17	2-methylbutanal	96-17-3	C_5_H_10_O	86.1	664.8	153.085	1.16143
18	3-methylbutanal	590-86-3	C_5_H_10_O	86.1	650.9	148.517	1.17545
19	3-methyl-2-Butenal	107-86-8	C_5_H_8_O	84.1	744.4	186.822	1.11091
20	octanol	111-87-5	C_8_H_18_O	130.2	1046.7	407.636	1.45537
21	2-ethyl-1-hexanol	104-76-7	C_8_H_18_O	130.2	1038.2	395.604	1.39794
22	1-octen-3-ol	3391-86-4	C_8_H_16_O	128.2	961.9	310.449	1.56875
23	(E)-3-hexen-1-ol	928-97-2	C_6_H_12_O	100.2	851.8	237.042	1.26041
24	4-methylpentanol	626-89-1	C_6_H_14_O	102.2	848.2	235.399	1.30886
25	4-hydroxy-4-methyl-2-pentanone	123-42-2	C_6_H_12_O_2_	116.2	838.4	230.893	1.51444
26	4-methyl-2-pentanol	108-11-2	C_6_H_14_O	102.2	754.4	191.766	1.25491
27	(Z)-2-pentenol	1576-95-0	C_5_H_10_O	86.1	773.7	201.776	1.45079
28	(E)-2-pentenal	1576-87-0	C_5_H_8_O	84.1	767.9	198.744	1.10396
29	1,2-dimethoxyethane	110-71-4	C_4_H_10_O_2_	90.1	649.6	148.092	1.13222
30	2,3-pentadione	600-14-6	C_5_H_8_O_2_	100.1	654.6	149.7	1.27829
31	2-heptanone (M)	110-43-0	C_7_H_14_O	114.2	889.8	255.489	1.26345
32	2-heptanone (D)	110-43-0	C_7_H_14_O	114.2	898.8	261.399	1.66038
33	4-methyl-3-penten-2-one	141-79-7	C_6_H_10_O	98.1	794.5	211.715	1.46891
34	1-penten-3-one	1629-58-9	C_5_H_8_O	84.1	681.8	158.862	1.32791
35	3-hydroxy-2-butanone	513-86-0	C_4_H_8_O_2_	88.1	734.7	182.086	1.35775
36	cyclopentanone	120-92-3	C_5_H_8_O	84.1	770.7	200.21	1.32899
37	2-ethyl-3,5-dimethylpyrazine	13925-07-0	C_8_H_12_N_2_	136.2	1074.5	450.057	1.22834
38	2-ethyl-5-methylpyrazine	13360-64-0	C_7_H_10_N_2_	122.2	1008.8	356.311	1.68005
39	2-methoxy-3-methylpyrazine	2847-30-5	C_6_H_8_N_2_O	124.1	986.4	331.832	1.16217
40	methylpyrazine	109-08-0	C_5_H_6_N_2_	94.1	816.9	221.302	1.08503
41	2-isobutyl-3-methoxypyrazine	24683-00-9	C_9_H_14_N_2_O	166.2	1176.1	645.781	1.81887
42	acetic acid, hexyl ester	142-92-7	C_8_H_16_O_2_	144.2	1009.5	357.195	1.40756
43	ethyl trans-2-butenoate	623-70-1	C_6_H_10_O_2_	114.1	841.1	232.123	1.18045
44	butyl acetate	123-86-4	C_6_H_12_O_2_	116.2	792.7	210.973	1.24106
45	butanoic acid methyl ester (M)	623-42-7	C_5_H_10_O_2_	102.1	716.9	173.741	1.13044
46	butanoic acid methyl ester (D)	623-42-7	C_5_H_10_O_2_	102.1	723.7	176.881	1.41501
47	2-pentanone	107-87-9	C_5_H_10_O	86.1	691.1	162.327	1.38112
48	ethyl acrylate	140-88-5	C_5_H_8_O_2_	100.1	704.3	168.064	1.41033
49	ethyl propanoate (M)	105-37-3	C_5_H_10_O_2_	102.1	703.6	167.745	1.48045
50	ethyl propanoate (D)	105-37-3	C_5_H_10_O_2_	102.1	704.5	168.17	1.16611
51	2,3-pentanedione	600-14-6	C_5_H_8_O_2_	100.1	625.4	140.48	1.28563
52	2,3-butanedione	431-03-8	C_4_H_6_O_2_	86.1	582.1	127.802	1.1808
53	acetic acid methyl ester	79-20-9	C_3_H_6_O_2_	74.1	522.1	112.125	1.1826
54	2-methylpropyl acetate	110-19-0	C_6_H_12_O_2_	116.2	791.3	210.377	1.62248
55	2-pentylfuran	3777-69-3	C_9_H_14_O	138.2	997.1	341.859	1.25701
56	2-Furanmethanol	98-00-0	C_5_H_6_O_2_	98.1	862	241.901	1.14061
57	allylacetic acid	591-80-0	C_5_H_8_O_2_	100.1	901.2	263.103	1.43113
58	2-methylpropanoic acid	79-31-2	C_4_H_8_O_2_	88.1	770.9	200.307	1.36084
59	Tetrahydrothiophene	110-01-0	C_4_H_8_S	88.2	816.9	221.302	1.33391
60	alpha-Terpinene	99-86-5	C_10_H_16_	136.2	1016.4	366.043	1.19378
61	Pyrrolidine	123-75-1	C_4_H_9_N	71.1	690.5	162.065	1.2915
62	2,6-dimethyl-pyridine	108-48-5	C_7_H_9_N	107.2	884.1	252.671	1.08859

CAS is the registration number of chemical substances by Chemical Abstracts Service. ^1^ represents the retention time in the capillary GC column; ^2^ represents the retention index calculated on an MXT-5 column using n-ketones C_4_-C_9_ as external standard; ^3^ represents the drift time in the drift tube.

**Table 2:** The GC-MS analyses of fatty acids in nutrient packs stored for 0 days by different manufacturers (mg/g).

	YYB-1A	YYB-2A	YYB-3A	YYB-4A	YYB-5A
C10:0	8.76 ± 0.57	–	–	–	8.64 ± 0.57
C12:0	23.22 ± 1.27 ^a^	–	18.54 ± 0.34 ^b^	–	22.95 ± 2.3 ^a^
C14:0	85.12 ± 2.06 ^a^	–	59.69 ± 0.8 ^b^	13.55 ± 0.56 ^c^	83.64 ± 2.72 ^a^
C14:1	4.78 ± 0.21	–	–	–	5.31 ± 0.59
C15:0	9.38 ± 0.34 ^a^	–	6.22 ± 0.14 ^b^	–	9.25 ± 0.25 ^a^
C16:0	1577.94 ± 39.09 ^a^	1208.29 ± 21.36 ^c^	1341.29 ± 20.37 ^b^	1344.17 ± 30.57 ^b^	1443.27 ± 43.66 ^a^
C16:1	14.18 ± 0.9 ^a^	3.75 ± 0.2 ^c^	10.72 ± 1.09 ^b^	4.9 ± 0.25 ^c^	14.32 ± 0.7 ^a^
C17:0	14.48 ± 0.02 ^a^	9.6 ± 0.61 ^c^	12.44 ± 0.17 ^b^	10.18 ± 0.69 ^c^	13.54 ± 0.72 ^ab^
C18:0	537.25 ± 13.39 ^a^	460.45 ± 7.03 ^bc^	451.89 ± 6.28 ^c^	420.22 ± 9.44 ^d^	482.12 ± 14.92 ^b^
C18:1n9c	1888.58 ± 47.77 ^a^	1669.97 ± 29.74 ^c^	1655.6 ± 24.31 ^c^	1487.73 ± 33.55 ^d^	1772.41 ± 58.71 ^b^
C18:2n6c	5233.62 ± 125.89 ^a^	4663.31 ± 81.66 ^b^	4314 ± 66.52 ^c^	4687.39 ± 104.83 ^b^	4756.86 ± 143.07 ^b^
C18:3n3	902.37 ± 20.6 ^a^	837.98 ± 20.19 ^bc^	724.84 ± 10.47 ^d^	793.14 ± 17.31 ^c^	844.84 ± 25.2 ^b^
C20:0	38.13 ± 1.29 ^a^	33.67 ± 1.56 ^b^	30.79 ± 0.57 ^c^	32.54 ± 0.74 ^bc^	32.48 ± 1.26 ^bc^
C20:1	16.75 ± 1.38 ^a^	14.26 ± 3.58 ^a^	11.55 ± 1.54 ^a^	12.58 ± 0.36 ^a^	11.33 ± 2.16 ^a^
C20:2	5.20 ± 0.45 ^a^	3.98 ± 0.62 ^a^	–	4.35 ± 0.16 ^a^	–
C22:0	55.08 ± 2.89 ^a^	46.33 ± 1.23 ^bc^	43.58 ± 0.43 ^c^	47.67 ± 2.99 ^bc^	49.23 ± 1.68 ^b^
C22:1n9	5.3 ± 0.52 ^a^	4.36 ± 0.45 ^a^	–	5.09 ± 0.85 ^a^	–
C23:0	7.58 ± 0.85 ^a^	5.24 ± 0.27 ^c^	6.87 ± 0.21 ^ab^	5.92 ± 0.71 ^bc^	6.95 ± 0.67 ^ab^
C24:0	17.33 ± 1.2 ^a^	15.72 ± 0.78 ^ab^	15.29 ± 0.4 ^ab^	14.76 ± 0.31 ^b^	16.24 ± 0.59 ^ab^

Data are presented as means ± standard deviation (*n* = 3). Samples marked with different letters within the same row show statistically significant differences (*p* < 0.05).

**Table 3:** The GC-MS analyses of fatty acids in nutrient packs stored for 90 days by different manufacturers (mg/g).

	YYB-1B	YYB-2B	YYB-3B	YYB-4B	YYB-5B
C10:0	–	–	–	–	12.48 ± 0.45
C12:0	15.08 ± 0.14 ^c^	–	28.78 ± 0.88 ^b^	–	31.63 ± 1.32 ^a^
C14:0	58.4 ± 0.48 ^c^	–	83.8 ± 3.15 ^b^	18.72 ± 0.43 ^d^	110.81 ± 3.93 ^a^
C14:1	–	–	4.98 ± 0.19	–	6.66 ± 1.01
C15:0	6.32 ± 0.41 ^c^	–	8.92 ± 0.25 ^b^	–	12.78 ± 0.25 ^a^
C16:0	1275.75 ± 15.89 ^cd^	1208.58 ± 16.96 ^d^	1369.07 ± 50.41 ^b^	1322.08 ± 12.38 ^bc^	1533.36 ± 58.91 ^a^
C16:1	9.91 ± 0.32 ^c^	4.15 ± 0.5 ^e^	13.88 ± 0.74 ^a^	5.16 ± 0.31 ^d^	18.7 ± 1.39 ^b^
C17:0	11.33 ± 0.31 ^c^	10.14 ± 0.44 ^c^	13.14 ± 0.39 ^b^	10.62 ± 0.31 ^c^	15 ± 0.84 ^a^
C18:0	426.06 ± 5.2 ^cd^	454.51 ± 6.56 ^bc^	459.67 ± 16.97 ^b^	422.56 ± 3.39 ^d^	518.35 ± 19.87 ^a^
C18:1n9c	1516.01 ± 17.7 ^c^	1661.15 ± 23.36 ^b^	1617.03 ± 58.37 ^bc^	1575.71 ± 15.22 ^bc^	1764.33 ± 66.27 ^a^
C18:2n6c	4317.87 ± 53.22 ^b^	4679.68 ± 64.51 ^a^	4118.66 ± 152.18 ^b^	4668.69 ± 43.4 ^a^	4641.05 ± 177.19 ^a^
C18:3n3	743.13 ± 8.96 ^c^	844.06 ± 11.94 ^a^	692.85 ± 25.44 ^d^	795.23 ± 6.78 ^b^	824.63 ± 32.44 ^ab^
C20:0	29.94 ± 0.79 ^bc^	33.33 ± 0.3 ^a^	28.08 ± 1.63 ^c^	33.85 ± 0.11 ^a^	31.79 ± 1.45 ^ab^
C20:1	9.29 ± 1.68 ^b^	12.68 ± 1.59 ^ab^	8.2 ± 1.92 ^b^	14.28 ± 0.3 ^a^	12.72 ± 2.64 ^ab^
C20:2	6.58 ± 0.97 ^a^	3.88 ± 0.04 ^b^	–	4.04 ± 0.37 ^b^	3.41 ± 0.1 ^b^
C22:0	43.23 ± 0.74 ^c^	43.73 ± 0.95 ^bc^	40.2 ± 0.94 ^d^	52.16 ± 0.86 ^a^	46.12 ± 1.89 ^b^
C22:1n9	4.22 ± 0.66 ^a^	4.37 ± 0.22 ^a^	3.95 ± 0.35 ^a^	5.07 ± 0.92 ^a^	5 ± 0.1 ^a^
C23:0	6.37 ± 0.24 ^a^	5.92 ± 0.47 ^a^	5.22 ± 0.68 ^a^	6.5 ± 0.3 ^a^	6.62 ± 1.16 ^a^
C24:0	13.54 ± 0.23 ^c^	15.98 ± 0.26 ^b^	13.01 ± 0.9 ^c^	18.66 ± 1.34 ^a^	16.01 ± 0.7 ^b^

Data are presented as means ± standard deviation (*n* = 3). Samples marked with different letters within the same row show statistically significant differences (*p* < 0.05).

## Data Availability

The original contributions presented in the study are included in the article, further inquiries can be directed to the corresponding author.
